# (*E*)-1-(4-Meth­oxy­phen­yl)-3-(2,4,6-trimeth­oxy­phen­yl)prop-2-en-1-one

**DOI:** 10.1107/S1600536811017788

**Published:** 2011-05-14

**Authors:** Yuepiao Cai, Zhankun Wang, Zhe Li, Meiling Zhang, Jianzhang Wu

**Affiliations:** a School of Pharmacy, Wenzhou Medical College, Wenzhou, Zhejiang Province 325035, People’s Republic of China; b Physical Education Department, Wenzhou Medical College, Wenzhou, Zhejiang Province 325035, People’s Republic of China; c Life Science College, Wenzhou Medical College, Wenzhou, Zhejiang Province 325035, People’s Republic of China

## Abstract

In the title compound, C_19_H_20_O_5_, the dihedral angle between the two aromatic rings is 18.23 (4)°. The crystal structure exhibits only weak C—H⋯π and C—H⋯O contacts between the mol­ecules.

## Related literature

For related structures, see: Wu *et al.* (2011 ▶); Peng *et al.* (2010 ▶); Huang *et al.* (2010 ▶); Zhao *et al.* (2010 ▶). For background and applications of chalcones, see: Wu *et al.* (2010 ▶, 2011) ▶; Liu *et al.* (2008 ▶); Zhao *et al.* (2010 ▶); Nielsen *et al.* (2005 ▶).
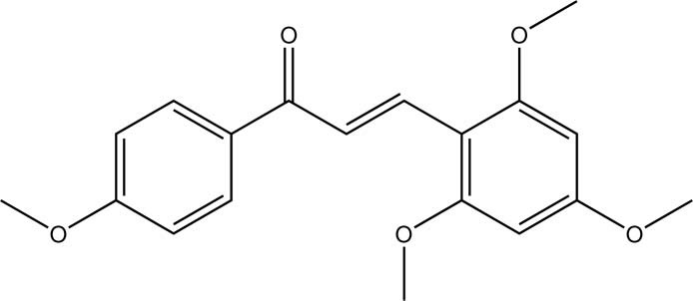


## Experimental

### 

#### Crystal data

C_19_H_20_O_5_
*M* *_r_* = 328.35
Orthorhombic, 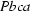

*a* = 7.3339 (6) Å
*b* = 16.8260 (14) Å
*c* = 26.677 (2) Å
*V* = 3291.9 (5) Å^3^
*Z* = 8
Mo *K*α radiation
μ = 0.10 mm^−1^
*T* = 133 K
0.35 × 0.33 × 0.31 mm

#### Data collection

Bruker SMART APEX CCD diffractometer
Absorption correction: multi-scan (*SADABS*; Bruker, 2001 ▶) *T*_min_ = 0.967, *T*_max_ = 0.971
22257 measured reflections
3593 independent reflections
3380 reflections with *I* > 2σ(*I*)
*R*_int_ = 0.020

#### Refinement

*R*[*F* ^2^ > 2σ(*F* ^2^)] = 0.037
*wR*(*F* ^2^) = 0.108
*S* = 1.02
3593 reflections
221 parameters
H-atom parameters constrained
Δρ_max_ = 0.22 e Å^−3^
Δρ_min_ = −0.24 e Å^−3^

### 

Data collection: *SMART* (Bruker, 2001 ▶); cell refinement: *SAINT* (Bruker, 2001 ▶); data reduction: *SAINT*; program(s) used to solve structure: *SHELXS97* (Sheldrick, 2008 ▶); program(s) used to refine structure: *SHELXL97* (Sheldrick, 2008 ▶); molecular graphics: *SHELXTL* (Sheldrick, 2008 ▶); software used to prepare material for publication: *SHELXTL*.

## Supplementary Material

Crystal structure: contains datablocks I, global. DOI: 10.1107/S1600536811017788/ff2009sup1.cif

Structure factors: contains datablocks I. DOI: 10.1107/S1600536811017788/ff2009Isup2.hkl

Supplementary material file. DOI: 10.1107/S1600536811017788/ff2009Isup3.cml

Additional supplementary materials:  crystallographic information; 3D view; checkCIF report

## Figures and Tables

**Table 1:** Hydrogen-bond geometry (Å, °) *Cg*1 and *Cg*2 are the centroids of the C1–C6 and C10–C15 rings, respectively.

*D*—H⋯*A*	*D*—H	H⋯*A*	*D*⋯*A*	*D*—H⋯*A*
C1—H1⋯*Cg*2^i^	0.95	2.89	3.6744 (12)	140
C4—H4⋯*Cg*1^ii^	0.95	2.94	3.6921 (12)	137
C17—H17a⋯*Cg*1^iii^	0.98	2.96	3.8913 (13)	159
C19—H19a⋯*Cg*2^iv^	0.98	2.82	3.4705 (13)	125
C16—H16c⋯O3^v^	0.98	2.51	3.4074 (16)	152
C18—H18a⋯O5^vi^	0.98	2.48	3.1578 (16)	126

Symmetry codes: (i) 
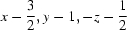
; (ii) 
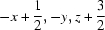
; (iii) 
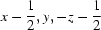
; (iv) 
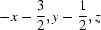
; (v) 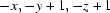; (vi) 
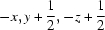
.

**Fig. 1.**
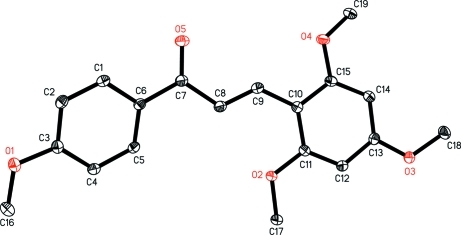
Molecular structure of the title compound. Displacement ellipsoids are drawn at the 30% probability level. All H atoms have been omitted for clarity.

## supplementary crystallographic information

### Comment

Chalcones, with the common skeleton of 1,3-diaryl-2-propen-1-one, are essential compounds in flavonoid biosynthesis in plants. They consist of two aromatic rings linked by a three-carbon α,β-unsaturated carbonyl system (Peng *et al.*, 2010; Huang *et al.*, 2010; Zhao *et al.*, 2010.).

Both natural and synthetic chalcones have active biological properties such as antiinflammatory, antitumoral, antioxidant, antibacterial (Wu *et al.* 2011; Liu *et al.*,  2008; Wu *et al.* 2010; Zhao, *et al.* 2010; Nielsen *et al.* 2005).

In order to investigate activity of chalcones, the title compound has been synthesised. Subsequently, its single-crystal X-ray study was carried out.

The dihedral angle between the two aromatic rings is 18.23 (4)°. There are weak C—H···π and C—H···O intermolecular interactions in the crystal structure. One of the methoxy groups in *ortho* position of 2,4,6-trimethoxyphenyl ring is slightly bent out of the ring plane [C14-C15-O4-C9 = 16.90 (16)°] while the other methoxy groups are almost coplanar with their parent ring planes [C-C-O-CH_3_ = 176.37 (10)°, 176.22 (9)° and -174.09 (10)°].

### Experimental

2,4,6-trimethoxybenzaldehyde (2 mmol) and 1-(4-dimethoxyphenyl)ethanone (2 mmol) were dissolved in ehanol (15 ml). The reaction temperature were about 305 K. The reaction was catalyzed by NaOH (20%, 5 drops). The reaction was monitored by thin-layer chromatography. After 10 h, 15 ml H_2_O was added and a yellow solid precipitated. The solid was washed with the mixture of water and cold ethanol, and dried. The pure compound was obtained by column chromatography on silica gel (yield: 67%). Single crystals of the compound were grown in a CH_2_Cl_2_/CH_3_CH_2_OH mixture (1:1 *v*/*v*) at 277 K.

### Refinement

All hydrogen atoms were positioned geometrically and refined using a riding model approximation, with C—H = 0.95–0.99 Å and with *U*_iso_(H) = 1.2–1.5 times *U*_eq_(C).

### Crystal data

**Table:**

C_19_H_20_O_5_	*F*(000) = 1392
*M**_r_* = 328.35	*D*_x_ = 1.325 Mg m^−^^3^
Orthorhombic, *P**b**c**a*	Mo *K*α radiation, λ = 0.71073 Å
Hall symbol: -P 2ac 2ab	Cell parameters from 9911 reflections
*a* = 7.3339 (6) Å	θ = 2.5–27.5°
*b* = 16.8260 (14) Å	µ = 0.10 mm^−^^1^
*c* = 26.677 (2) Å	*T* = 133 K
*V* = 3291.9 (5) Å^3^	Block, colourless
*Z* = 8	0.35 × 0.33 × 0.31 mm

### Data collection

**Table:**

Bruker SMART APEX CCD diffractometer	3593 independent reflections
Radiation source: fine-focus sealed tube	3380 reflections with *I* > 2σ(*I*)
graphite	*R*_int_ = 0.020
φ and ω scans	θ_max_ = 27.0°, θ_min_ = 1.5°
Absorption correction: multi-scan (*SADABS*; Bruker, 2001)	*h* = −9→8
*T*_min_ = 0.967, *T*_max_ = 0.971	*k* = −21→21
22257 measured reflections	*l* = −34→33

### Refinement

**Table:**

Refinement on *F*^2^	Primary atom site location: structure-invariant direct methods
Least-squares matrix: full	Secondary atom site location: difference Fourier map
*R*[*F*^2^ > 2σ(*F*^2^)] = 0.037	Hydrogen site location: inferred from neighbouring sites
*wR*(*F*^2^) = 0.108	H-atom parameters constrained
*S* = 1.02	*w* = 1/[σ^2^(*F*_o_^2^) + (0.0656*P*)^2^ + 1.0603*P*] where *P* = (*F*_o_^2^ + 2*F*_c_^2^)/3
3593 reflections	(Δ/σ)_max_ < 0.001
221 parameters	Δρ_max_ = 0.22 e Å^−^^3^
0 restraints	Δρ_min_ = −0.24 e Å^−^^3^

### Special details

**Table:**

Geometry. All e.s.d.'s (except the e.s.d. in the dihedral angle between two l.s. planes) are estimated using the full covariance matrix. The cell e.s.d.'s are taken into account individually in the estimation of e.s.d.'s in distances, angles and torsion angles; correlations between e.s.d.'s in cell parameters are only used when they are defined by crystal symmetry. An approximate (isotropic) treatment of cell e.s.d.'s is used for estimating e.s.d.'s involving l.s. planes.
Refinement. Refinement of *F*^2^ against ALL reflections. The weighted *R*-factor *wR* and goodness of fit *S* are based on *F*^2^, conventional *R*-factors *R* are based on *F*, with *F* set to zero for negative *F*^2^. The threshold expression of *F*^2^ > σ(*F*^2^) is used only for calculating *R*-factors(gt) *etc*. and is not relevant to the choice of reflections for refinement. *R*-factors based on *F*^2^ are statistically about twice as large as those based on *F*, and *R*- factors based on ALL data will be even larger.

### Fractional atomic coordinates and isotropic or equivalent isotropic displacement parameters (Å^2^)

**Table:**

	*x*	*y*	*z*	*U*_iso_*/*U*_eq_	
O1	−0.11440 (12)	0.09104 (5)	0.52298 (3)	0.0306 (2)	
O2	0.09328 (11)	0.52918 (4)	0.42318 (3)	0.02358 (18)	
O3	0.15756 (13)	0.77278 (5)	0.33786 (3)	0.0297 (2)	
O4	−0.01580 (13)	0.53324 (5)	0.24951 (3)	0.0297 (2)	
O5	−0.12136 (16)	0.28670 (6)	0.32160 (3)	0.0432 (3)	
C1	−0.17359 (15)	0.18362 (6)	0.40208 (4)	0.0236 (2)	
H1	−0.2334	0.1716	0.3714	0.028*	
C2	−0.18310 (15)	0.13044 (6)	0.44147 (4)	0.0249 (2)	
H2	−0.2496	0.0823	0.4378	0.030*	
C3	−0.09526 (15)	0.14727 (6)	0.48666 (4)	0.0231 (2)	
C4	0.00426 (15)	0.21720 (6)	0.49199 (4)	0.0237 (2)	
H4	0.0660	0.2285	0.5225	0.028*	
C5	0.01209 (15)	0.27031 (6)	0.45199 (4)	0.0228 (2)	
H5	0.0797	0.3181	0.4556	0.027*	
C6	−0.07673 (14)	0.25507 (6)	0.40679 (4)	0.0217 (2)	
C7	−0.07394 (16)	0.31067 (7)	0.36306 (4)	0.0255 (2)	
C8	−0.01821 (16)	0.39334 (7)	0.37223 (4)	0.0250 (2)	
H8	0.0155	0.4091	0.4051	0.030*	
C9	−0.01402 (15)	0.44698 (7)	0.33494 (4)	0.0238 (2)	
H9	−0.0486	0.4273	0.3029	0.029*	
C10	0.03548 (14)	0.53061 (6)	0.33628 (4)	0.0212 (2)	
C11	0.08965 (14)	0.57253 (6)	0.37997 (4)	0.0206 (2)	
C12	0.13343 (15)	0.65221 (6)	0.37886 (4)	0.0227 (2)	
H12	0.1726	0.6784	0.4085	0.027*	
C13	0.11981 (15)	0.69401 (6)	0.33384 (4)	0.0231 (2)	
C14	0.07144 (15)	0.65615 (7)	0.28945 (4)	0.0239 (2)	
H14	0.0653	0.6847	0.2588	0.029*	
C15	0.03215 (15)	0.57507 (7)	0.29121 (4)	0.0226 (2)	
C16	−0.0194 (2)	0.10198 (8)	0.56923 (5)	0.0365 (3)	
H16A	0.1116	0.1067	0.5626	0.055*	
H16B	−0.0416	0.0563	0.5912	0.055*	
H16C	−0.0631	0.1505	0.5856	0.055*	
C17	0.15775 (17)	0.56770 (7)	0.46741 (4)	0.0274 (2)	
H17A	0.2826	0.5866	0.4619	0.041*	
H17B	0.1563	0.5300	0.4954	0.041*	
H17C	0.0787	0.6130	0.4752	0.041*	
C18	0.1300 (2)	0.82163 (7)	0.29468 (5)	0.0364 (3)	
H18A	0.2094	0.8034	0.2675	0.055*	
H18B	0.1595	0.8769	0.3029	0.055*	
H18C	0.0024	0.8181	0.2840	0.055*	
C19	0.02210 (17)	0.56754 (7)	0.20163 (4)	0.0286 (3)	
H19A	−0.0531	0.6151	0.1970	0.043*	
H19B	−0.0059	0.5289	0.1752	0.043*	
H19C	0.1513	0.5821	0.1998	0.043*	

### Atomic displacement parameters (Å^2^)

**Table:**

	*U*^11^	*U*^22^	*U*^33^	*U*^12^	*U*^13^	*U*^23^
O1	0.0364 (5)	0.0276 (4)	0.0278 (4)	−0.0038 (3)	−0.0007 (3)	0.0050 (3)
O2	0.0312 (4)	0.0216 (4)	0.0179 (4)	−0.0018 (3)	−0.0029 (3)	−0.0002 (3)
O3	0.0428 (5)	0.0211 (4)	0.0253 (4)	−0.0041 (3)	−0.0032 (3)	0.0026 (3)
O4	0.0398 (5)	0.0312 (4)	0.0182 (4)	−0.0090 (4)	−0.0033 (3)	0.0003 (3)
O5	0.0722 (7)	0.0338 (5)	0.0238 (4)	−0.0168 (5)	−0.0113 (4)	−0.0003 (3)
C1	0.0224 (5)	0.0244 (5)	0.0240 (5)	−0.0017 (4)	−0.0004 (4)	−0.0042 (4)
C2	0.0225 (5)	0.0223 (5)	0.0299 (6)	−0.0043 (4)	0.0014 (4)	−0.0021 (4)
C3	0.0218 (5)	0.0223 (5)	0.0251 (5)	0.0029 (4)	0.0040 (4)	0.0012 (4)
C4	0.0238 (5)	0.0245 (5)	0.0229 (5)	0.0009 (4)	−0.0014 (4)	−0.0034 (4)
C5	0.0238 (5)	0.0203 (5)	0.0241 (5)	−0.0022 (4)	0.0005 (4)	−0.0034 (4)
C6	0.0220 (5)	0.0210 (5)	0.0222 (5)	−0.0001 (4)	0.0021 (4)	−0.0032 (4)
C7	0.0296 (6)	0.0251 (5)	0.0219 (5)	−0.0029 (4)	−0.0009 (4)	−0.0017 (4)
C8	0.0289 (5)	0.0245 (5)	0.0218 (5)	−0.0022 (4)	−0.0001 (4)	−0.0026 (4)
C9	0.0240 (5)	0.0245 (5)	0.0228 (5)	−0.0017 (4)	−0.0008 (4)	−0.0029 (4)
C10	0.0192 (5)	0.0228 (5)	0.0216 (5)	0.0000 (4)	0.0004 (4)	0.0001 (4)
C11	0.0179 (5)	0.0243 (5)	0.0195 (5)	0.0020 (4)	0.0011 (4)	0.0011 (4)
C12	0.0238 (5)	0.0236 (5)	0.0207 (5)	0.0001 (4)	0.0003 (4)	−0.0015 (4)
C13	0.0218 (5)	0.0216 (5)	0.0258 (5)	−0.0006 (4)	0.0018 (4)	0.0007 (4)
C14	0.0239 (5)	0.0270 (5)	0.0206 (5)	−0.0003 (4)	−0.0003 (4)	0.0026 (4)
C15	0.0202 (5)	0.0275 (5)	0.0202 (5)	−0.0007 (4)	−0.0007 (4)	−0.0013 (4)
C16	0.0475 (8)	0.0368 (7)	0.0252 (6)	−0.0010 (6)	−0.0025 (5)	0.0057 (5)
C17	0.0367 (6)	0.0252 (5)	0.0204 (5)	−0.0020 (5)	−0.0060 (4)	−0.0009 (4)
C18	0.0507 (8)	0.0267 (6)	0.0319 (6)	−0.0080 (5)	−0.0088 (6)	0.0085 (5)
C19	0.0315 (6)	0.0354 (6)	0.0189 (5)	−0.0007 (5)	−0.0016 (4)	0.0014 (4)

### Geometric parameters (Å, °)

**Table:**

O1—C3	1.3615 (13)	C9—C10	1.4538 (15)
O1—C16	1.4287 (15)	C9—H9	0.9500
O2—C11	1.3643 (12)	C10—C15	1.4163 (14)
O2—C17	1.4269 (12)	C10—C11	1.4191 (14)
O3—C13	1.3584 (13)	C11—C12	1.3789 (15)
O3—C18	1.4294 (13)	C12—C13	1.3954 (15)
O4—C15	1.3623 (13)	C12—H12	0.9500
O4—C19	1.4290 (13)	C13—C14	1.3907 (15)
O5—C7	1.2275 (14)	C14—C15	1.3951 (16)
C1—C2	1.3818 (15)	C14—H14	0.9500
C1—C6	1.4021 (15)	C16—H16A	0.9800
C1—H1	0.9500	C16—H16B	0.9800
C2—C3	1.3959 (16)	C16—H16C	0.9800
C2—H2	0.9500	C17—H17A	0.9800
C3—C4	1.3919 (15)	C17—H17B	0.9800
C4—C5	1.3931 (15)	C17—H17C	0.9800
C4—H4	0.9500	C18—H18A	0.9800
C5—C6	1.3941 (15)	C18—H18B	0.9800
C5—H5	0.9500	C18—H18C	0.9800
C6—C7	1.4955 (15)	C19—H19A	0.9800
C7—C8	1.4703 (15)	C19—H19B	0.9800
C8—C9	1.3435 (15)	C19—H19C	0.9800
C8—H8	0.9500		
			
C3—O1—C16	118.34 (9)	C11—C12—C13	119.46 (10)
C11—O2—C17	117.53 (8)	C11—C12—H12	120.3
C13—O3—C18	117.94 (9)	C13—C12—H12	120.3
C15—O4—C19	118.10 (9)	O3—C13—C14	124.49 (10)
C2—C1—C6	120.84 (10)	O3—C13—C12	114.15 (9)
C2—C1—H1	119.6	C14—C13—C12	121.36 (10)
C6—C1—H1	119.6	C13—C14—C15	118.16 (10)
C1—C2—C3	120.13 (10)	C13—C14—H14	120.9
C1—C2—H2	119.9	C15—C14—H14	120.9
C3—C2—H2	119.9	O4—C15—C14	122.07 (10)
O1—C3—C4	124.67 (10)	O4—C15—C10	115.14 (9)
O1—C3—C2	115.22 (10)	C14—C15—C10	122.78 (10)
C4—C3—C2	120.11 (10)	O1—C16—H16A	109.5
C3—C4—C5	119.05 (10)	O1—C16—H16B	109.5
C3—C4—H4	120.5	H16A—C16—H16B	109.5
C5—C4—H4	120.5	O1—C16—H16C	109.5
C4—C5—C6	121.69 (10)	H16A—C16—H16C	109.5
C4—C5—H5	119.2	H16B—C16—H16C	109.5
C6—C5—H5	119.2	O2—C17—H17A	109.5
C5—C6—C1	118.16 (10)	O2—C17—H17B	109.5
C5—C6—C7	123.58 (10)	H17A—C17—H17B	109.5
C1—C6—C7	118.26 (9)	O2—C17—H17C	109.5
O5—C7—C8	122.65 (10)	H17A—C17—H17C	109.5
O5—C7—C6	119.56 (10)	H17B—C17—H17C	109.5
C8—C7—C6	117.76 (9)	O3—C18—H18A	109.5
C9—C8—C7	121.24 (10)	O3—C18—H18B	109.5
C9—C8—H8	119.4	H18A—C18—H18B	109.5
C7—C8—H8	119.4	O3—C18—H18C	109.5
C8—C9—C10	129.63 (10)	H18A—C18—H18C	109.5
C8—C9—H9	115.2	H18B—C18—H18C	109.5
C10—C9—H9	115.2	O4—C19—H19A	109.5
C15—C10—C11	116.08 (9)	O4—C19—H19B	109.5
C15—C10—C9	119.08 (9)	H19A—C19—H19B	109.5
C11—C10—C9	124.83 (9)	O4—C19—H19C	109.5
O2—C11—C12	122.24 (9)	H19A—C19—H19C	109.5
O2—C11—C10	115.70 (9)	H19B—C19—H19C	109.5
C12—C11—C10	122.06 (10)		
			
C6—C1—C2—C3	−0.21 (16)	C10—C11—O2—C17	176.22 (9)
C16—O1—C3—C4	−3.10 (16)	C15—C10—C11—O2	−179.71 (9)
C2—C3—O1—C16	176.37 (10)	C9—C10—C11—O2	−0.21 (15)
C1—C2—C3—O1	179.65 (10)	C15—C10—C11—C12	1.10 (15)
C1—C2—C3—C4	−0.85 (16)	C9—C10—C11—C12	−179.40 (10)
O1—C3—C4—C5	−179.52 (10)	O2—C11—C12—C13	−177.37 (10)
C2—C3—C4—C5	1.03 (16)	C10—C11—C12—C13	1.76 (16)
C3—C4—C5—C6	−0.17 (16)	C18—O3—C13—C14	5.92 (17)
C4—C5—C6—C1	−0.86 (16)	C12—C13—O3—C18	−174.09 (10)
C4—C5—C6—C7	179.29 (10)	C11—C12—C13—O3	176.86 (10)
C2—C1—C6—C5	1.04 (16)	C11—C12—C13—C14	−3.16 (16)
C2—C1—C6—C7	−179.09 (10)	O3—C13—C14—C15	−178.47 (10)
C5—C6—C7—O5	164.74 (12)	C12—C13—C14—C15	1.56 (17)
C1—C6—C7—O5	−15.12 (16)	C14—C15—O4—C19	16.90 (16)
C5—C6—C7—C8	−16.87 (16)	C19—O4—C15—C10	−164.29 (10)
C1—C6—C7—C8	163.27 (10)	C13—C14—C15—O4	−179.79 (10)
O5—C7—C8—C9	−1.00 (19)	C13—C14—C15—C10	1.49 (17)
C6—C7—C8—C9	−179.34 (10)	C11—C10—C15—O4	178.43 (9)
C7—C8—C9—C10	179.64 (11)	C9—C10—C15—O4	−1.10 (15)
C8—C9—C10—C15	178.89 (11)	C11—C10—C15—C14	−2.77 (16)
C8—C9—C10—C11	−0.59 (19)	C9—C10—C15—C14	177.70 (10)
C17—O2—C11—C12	−4.60 (15)		

### Hydrogen-bond geometry (Å, °)

**Table:**

Cg1 and Cg2 are the centroids of the C1–C6 and C10–C15 rings, respectively.

**Table:**

*D*—H···*A*	*D*—H	H···*A*	*D*···*A*	*D*—H···*A*
C1—H1···Cg2^i^	0.95	2.89	3.6744 (12)	140
C4—H4···Cg1^ii^	0.95	2.94	3.6921 (12)	137
C17—H17a···Cg1^iii^	0.98	2.96	3.8913 (13)	159
C19—H19a···Cg2^iv^	0.98	2.82	3.4705 (13)	125
C16—H16c···O3^v^	0.98	2.51	3.4074 (16)	152
C18—H18a···O5^vi^	0.98	2.48	3.1578 (16)	126

Symmetry codes: (i) *x*−3/2, *y*−1, −*z*−1/2; (ii) −*x*+1/2, −*y*, *z*+3/2; (iii) *x*−1/2, *y*, −*z*−1/2; (iv) −*x*−3/2, *y*−1/2, *z*; (v) −*x*, −*y*+1, −*z*+1; (vi) −*x*, *y*+1/2, −*z*+1/2.
